# Myeloid cell leukemia-1: a formidable barrier to anticancer therapeutics and the quest of targeting it

**DOI:** 10.37349/etat.2022.00083

**Published:** 2022-05-24

**Authors:** Prasad Sulkshane, Tanuja Teni

**Affiliations:** 1 Glickman Laboratory, Faculty of Biology, Technion-Israel Institute of Technology, Haifa 3200003, Israel; 2 Teni Laboratory, Advanced Centre for Treatment, Research and Education in Cancer (ACTREC), Tata Memorial Centre, Kharghar, Navi Mumbai 410210, India; 3 Homi Bhabha National Institute, Training School Complex, Mumbai 400094, India; Regina Elena National Cancer Institute, Italy

**Keywords:** Myeloid cell leukemia-1, B cell lymphoma-2, mitochondrial outer membrane permeabilization, Bcl-2 homology 3 mimetic, therapy resistance, myeloid cell leukemia-1 inhibitor

## Abstract

The antiapoptotic B cell lymphoma-2 (Bcl-2) family members are apical regulators of the intrinsic pathway of apoptosis that orchestrate mitochondrial outer membrane permeabilization (MOMP) through interactions with their proapoptotic counterparts. Overexpression of antiapoptotic Bcl-2 family proteins has been linked to therapy resistance and poor prognosis in diverse cancers. Among the antiapoptotic Bcl-2 family members, predominant overexpression of the prosurvival myeloid cell leukemia-1 (Mcl-1) has been reported in a myriad of hematological malignancies and solid tumors, contributing to therapy resistance and poor outcomes, thus making it a potential druggable target. The unique structure of Mcl-1 and its complex regulatory mechanism makes it an adaptive prosurvival switch that ensures tumor cell survival despite therapeutic intervention. This review focusses on diverse mechanisms adopted by tumor cells to maintain sustained elevated levels of Mcl-1 and how high Mcl-1 levels contribute to resistance in conventional as well as targeted therapies. Moreover, recent developments in the Mcl-1-targeted therapeutics and the underlying challenges and considerations in designing novel Mcl-1 inhibitors are also discussed.

## Introduction

Fine regulation of apoptosis is central to the normal development and homeostasis in metazoans, dysregulation of which leads to abnormal development and cancer [1]. Research over the past several decades has provided a deep insight into the intricate mechanistic details of the intrinsic (mitochondrial) pathway of apoptosis. The B cell lymphoma-2 (Bcl-2) family proteins, which localize to the mitochondrial outer membrane (MOM) are the pivotal regulators of the intrinsic pathway of apoptosis which essentially involves MOM permeabilization (MOMP) and leads to the release of soluble proteins such as cytochrome C from the mitochondrial intermembrane space to the cytosol. Once in the cytosol, cytochrome C triggers a cascade of caspase activation which executes the cell death sequence and unleashes the cellular hallmarks of apoptosis [2]. The Bcl-2 family is composed of an elaborate set of proteins that are categorized into two major subfamilies: the antiapoptotic proteins, which prevent the MOMP and the proapoptotic proteins that promote the MOMP. The categorization is further based on the composition of the number of different Bcl-2 homology (BH) domains. The antiapoptotic proteins [Bcl-2, Bcl-extra large (Bcl-xl), Bcl-2 like 2 (Bcl-2L2/Bcl-w), Bcl-2-related protein A1 (Bcl-2A1/Bfl-1), myeloid cell leukemia-1 (Mcl-1)] typically consist of multiple BH domains (BH1 through BH4). The proapoptotic subfamily is further divided into multidomain members [Bcl-2-associated x protein (Bax), Bcl-2 antagonist killer 1 (Bak), Bcl-2 related ovarian killer (Bok)] and BH3-only members that harbor only the BH3 domain. The BH3-only proteins are further categorized as “Sensitizer” [Bcl-2 interacting killer (Bik), Bcl-2 antagonist of cell death (Bad), Bcl-2 modifying factor (Bmf), Harakiri (Hrk), Noxa] and “Activator” [Bcl-2 interacting domain death agonist (Bid), Bcl-2-interacting mediator of cell death (Bim), p53-upregulated modulator of apoptosis (Puma)] [3]. Upon receiving apoptotic stimulus, the Sensitizer BH3-only proteins interact with the antiapoptotic members to neutralize them. Simultaneously, the Activator BH3-only proapoptotic members interact with the multidomain proapoptotic proteins—Bak, Bax, bringing about conformational changes in them and thereby triggering their oligomerization to form hydrophilic channels in the MOM for cytochrome C release. Thus the fine-tuning of the relative levels and interactions between the antiapoptotic and proapoptotic Bcl-2 family members regulate the MOMP [4].

## Mcl-1 is a fine-tuned cell fate switch that malfunctions in cancer

Among the antiapoptotic Bcl-2 family members, Mcl-1 unarguably has the most dramatic mode of regulation [5]. *Mcl-1* gene is located on chromosome 1q21.2 and encodes for four BH domains—BH1 through BH4, a C-terminal transmembrane (TM) domain, and the N-terminal highly unstructured region consisting of regulatory motifs [6, 7]. Mcl-1 expression and function are tightly regulated at multiple levels including genomic, transcriptional, post-transcriptional, translational, post-translational, and through protein-protein interactions [8]. One of the most unique features of Mcl-1 is its rapid turnover and short half-life which makes it an ideal cell fate switch to rapidly respond to environmental cues [9]. Evidently, Mcl-1 is critical during early embryonic development and for the survival of the hematopoietic lineage in mammals [10, 11].

**Figure 1.**
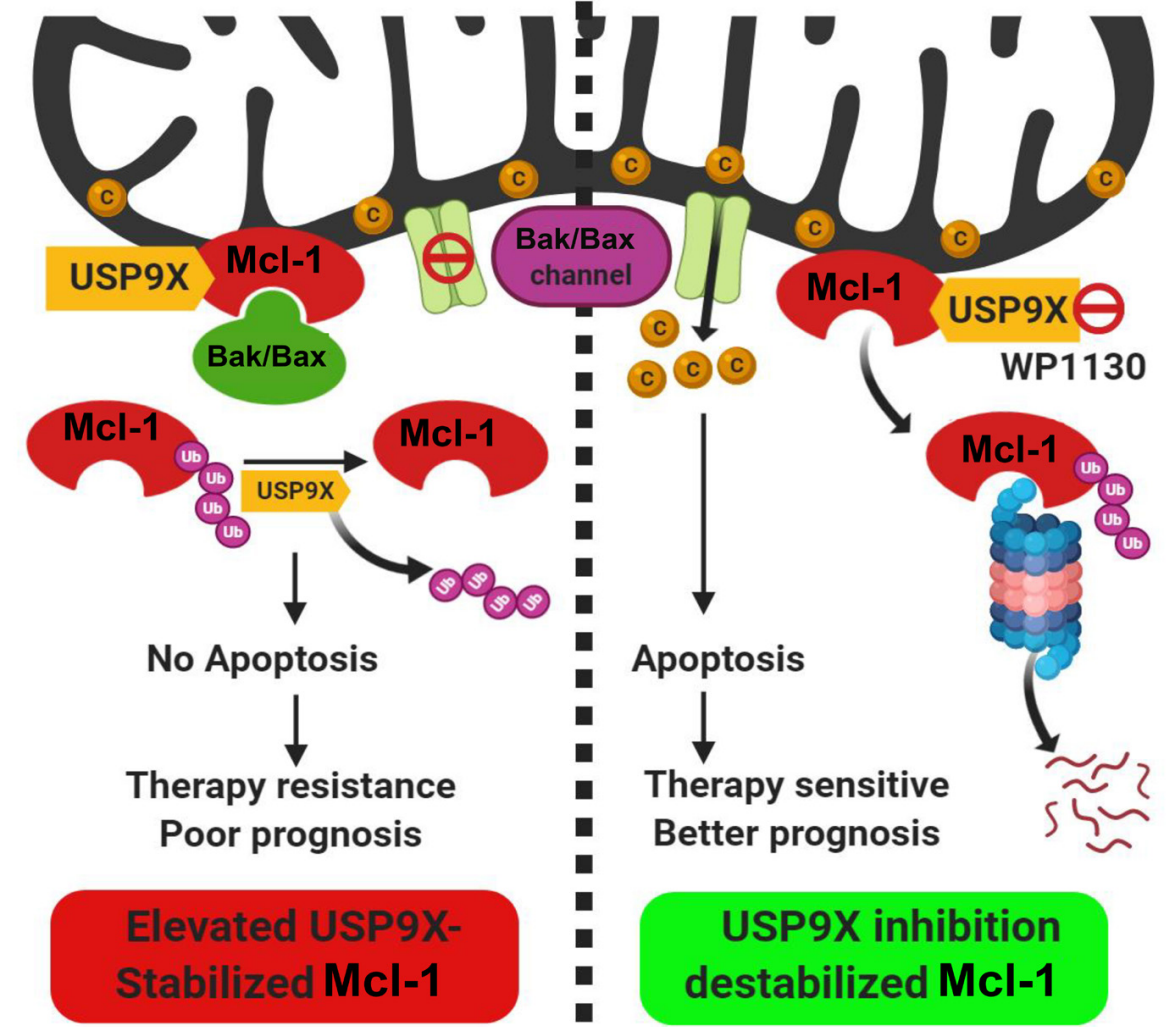
Posttranslational regulation of Mcl-1 protein stability by the deubiquitinase USP9X. The deubiquitinase USP9X interacts with Mcl-1 protein which localizes to the mitochondrial outer membrane. USP9X deubiquitinates and stabilizes Mcl-1 protein which in turn through interaction with BAK/BAX proteins, prevents their oligomerization. In the tumors with elevated USP9X expression, therefore apoptosis is blocked which culminates into therapy resistance and poor prognosis. Pharmacological inhibition of USP9X (with a small molecule inhibitor WP1130) causes ubiquitin-proteasome-dependent degradation of Mcl-1, causing enhanced apoptosis and better therapeutic outcome. C: cytochrome C; Ub: ubiquitin. (Figure created with BioRender.com)

Despite its tight regulatory circuit, diverse tumors often exhibit overexpression of Mcl-1 protein [12]. The *Mcl-1* gene is located on chromosome 1q21, a region that is frequently subjected to alterations in a myriad of solid tumors and hematological malignancies [6]. Mcl-1 protein overexpression has been demonstrated in diverse cancers ranging from solid tumors including oral cancers as reported by our group [13–15] to hematological malignancies [16–18]. Interestingly, an expression survey of Bcl-2 family proteins across an array of cancer cell lines of diverse lineages demonstrated a predominant overexpression of Mcl-1, underscoring its role as a key prosurvival protein for tumor cells [19]. The dysregulation of Mcl-1 may occur at multiple levels so as to maintain its sustained elevated levels to ensure tumor cell survival [20]. A genome-wide somatic copy number (SCN) and genome-wide association studies (GWAS) of clinical samples revealed the SCN amplification of the *Mcl-1* gene and its correlation with the increased survival potential of 26 diverse tumors [21]. For enhanced biosynthesis of Mcl-1 protein, tumor cells rely on chromosomal alterations that allow increased transcription of the *Mcl-1* gene such as through increased accessibility of its promoter [22], increased gene dosage through chromosomal translocation events that involve the *Mcl-1* gene [23, 24], enhanced translation of the Mcl-1 messenger RNA (mRNA) [25] and increased stabilization of Mcl-1 mRNA by regulation of microRNAs (miRNAs) targeting it [26–28]. In oral cancers, our laboratory has reported primarily increased levels of Mcl-1 transcripts which correlated well with the overall increase in the Mcl-1 protein levels [15]. At the post-transcriptional level, differential mRNA splicing can generate three alternate isoforms of Mcl-1 protein with opposing functions: the predominant long isoform [Mcl-1_L_: antiapoptotic; consists of 350 amino acid (aa) residues] and the short isoform (Mcl-1_S_; consists of 271 aa residues) and extra short isoform (Mcl-1_ES_; consists of 196 aa residues), both of which are proapoptotic in nature [8]. Alternative splicing of Mcl-1 mRNA leads to the exclusion of exon 2 that generates Mcl-1_S_ with missing BH1, BH2, and TM domains [29]. Subsequently, an extra short transcript variant of Mcl-1 (Mcl-1_ES_) was characterized which results from splicing within the first exon causing N-terminal truncation missing the proline-glutamic acid-serine-threonine (PEST) domains [30]. Both Mcl-1_S_ and Mcl-1_ES_ can interact with the antiapoptotic Mcl-1_L_, counteract its function and thereby trigger apoptosis. As a testimony of the prognostic potential of the Mcl-1 transcript variants, we have demonstrated that the antiapoptotic Mcl-1 long isoform transcript variant was particularly overexpressed in oral cancers and was also associated with poor prognosis and chemoresistance [31]. The regulation of Mcl-1 at the posttranslational level is perhaps the most dramatic among the antiapoptotic Bcl-2 family proteins. The N-terminal unstructured regulatory domain of Mcl-1 has several phosphorylation and ubiquitination sites organized into “PEST domains” and is responsible for its rapid turnover through proteasome-dependent degradation. This region of Mcl-1 is subject to ubiquitination by E3 ligases Mcl-1 ubiquitin ligase E3 (MULE) [32], Skp1, Cdc53/Cul1, F-box protein beta-transducin repeat containing protein (SCF^β-TrCP^) [33], anaphase promoting complex/cyclosome-cell division cycle protein 20 homolog (APC/C^cdc20^) [34] and F-box and tryptophan-aspartic acid (WD) repeat-containing protein 7 (FBX^FBW7^) [35]. Mcl-1 is protected from proteasomal degradation by a deubiquitinase ubiquitin specific peptidase 9 X-linked (USP9X) which removes the ubiquitin chains conjugated onto the Mcl-1 (Figure 1) [36, 37]. Our recent studies have demonstrated the prognostic potential of USP9X in oral cancers. We showed that its overexpression contributes to the stabilization of Mcl-1 protein from early to the late stages of oral tumorigenesis and also to therapy resistance leading to poor prognosis. Moreover, pharmacological inhibition of USP9X potently induced cell death in oral cancer cells through rapid degradation of Mcl-1 [37]. More recently, the deubiquitinases USP13 and USP7 have also been shown to contribute to Mcl-1 stabilization and drive tumorigenesis [38, 39]. Thus, the complex multimodal upregulation of prosurvival Mcl-1 ensures tumor cell viability and thereby contributes to tumorigenesis.

### Mcl-1 overexpression in cancer is linked to resistance against conventional therapeutics and poor prognosis

A marked overexpression of Mcl-1 more than any other Bcl-2 family member proteins has been documented in a wide variety of human cancer cell lines [19]. As discussed earlier, Mcl-1 overexpression has been associated with poor prognosis in a wide variety of cancers irrespective of their lineages [40–46]. The principal factor promoting tumor cell survival is the acquired virtue of therapy resistance. A number of reports have demonstrated the importance of Mcl-1 for chemoresistance in cancer cells [47–49] (Table 1). Mcl-1 overexpression has been linked to the acquired cisplatin resistance in cancer cells belonging to various lineages and that its targeted downregulation or pharmacological inhibition sensitized these cells to cisplatin [31, 50 –52]. Particularly, our studies in oral cancers demonstrated the prognostic significance of the antiapoptotic splice variant of Mcl-1 (Mcl-1_L_) with Cisplatin resistance and that its targeted downregulation or small molecule-mediated inhibition restored the sensitivity of oral cancer cells towards Cisplatin [31]. Furthermore, Mcl-1 overexpression is also associated with acquired radioresistance in cancer cells, including in oral cancer cells as shown in our studies [15, 53–57]. It is thus evident that irrespective of the kind of conventional therapeutic agent, cancer cells tend to upregulate Mcl-1 to block cell death ensuring cell survival.

**Table 1.** Pathological impact of Mcl-1 overexpression in cancers

**Type of cancer**	**Pathological impact of Mcl-1 overexpression**	**References**
Prostate	Resistance to therapies and apoptosis	[48, 126]
Breast	Resistance to therapies and apoptosis, contributes to metastasis	[42, 68]
Ovarian	Therapy resistance, poor prognosis	[43, 127]
Renal	Blocks apoptosis	[128]
Lung	Promotes survival and drives lung cancer progression	[14, 129]
Colon	Resistance to targeted therapies and apoptosis	[66, 130]
Pancreatic	Resistance to therapies and apoptosis	[131, 132]
Head and neck	Resistance to therapies, tumor progression, poor prognosis	[15, 133]
Melanoma	Resistance to apoptosis	[105, 134]
MM	Disease relapse, poor prognosis	[135, 136]
AML	Therapy resistance, disease relapse	[18, 137]
NHL	Disease progression, high-grade lymphoma	[138, 139]

MM: multiple myeloma; AML: acute myeloid leukemia; NHL: non-Hodgkin lymphoma

### Mcl-1 resists targeted therapies to serve as an adaptive driver of cancer cell survival

Mcl-1 is known to confer resistance against targeted therapies in addition to conventional therapies [58]. In a comprehensive study encompassing 21 cancer cell lines belonging to diverse lineages, each characterized by a distinct oncoprotein [B-Raf proto-oncogene (BRAF), CKIT, epidermal growth factor receptor (EGFR), MET, anaplastic lymphoma kinase (ALK), erb-b2 receptor tyrosine kinase 2 (ERBB2)], Mcl-1 was found to be specifically important for their survival when treated with specific inhibitors of the responsible oncoproteins. It was found that these multiple targeted therapies caused destabilization of the Noxa mRNA which further led to Mcl-1 stabilization. Downregulation of Mcl-1 but not other antiapoptotic Bcl-2 family proteins potentiated the cell killing efficacy of these specific inhibitors, suggesting that regardless of the tumor lineage, the responsible oncoprotein or the corresponding targeted inhibitors, Mcl-1 was a common resistance factor [59]. Since Noxa controls the MULE E3 ligase-dependent ubiquitination of Mcl-1, depletion of Noxa mRNA following treatment with the targeted therapies results in rapid stabilization of Mcl-1 protein [60]. Mcl-1 was found to be responsible for BRAF and mitogen-activated extracellular signal-regulated kinase (ERK) kinase (MEK)1/2 inhibitor resistance in melanoma setting and that Mcl-1 inhibition delayed the acquired BRAF/MEK-inhibitor resistance and thereby increased the efficacy of ERK1/2 inhibitor [61]. Mcl-1 upregulation was found to be the main reason for acquired resistance to the third-generation EGFR inhibitor AZD9291 in EGFR-mutant non-small cell lung cancer cell lines. However, when AZD9291 was combined with the MEK/ERK inhibitor GSK212, Bim levels were elevated, causing Mcl-1 degradation and ultimately leading to enhanced sensitization of the cells to the EGFR inhibitor [62]. Mutations in the tumor suppressor FBW7 causes elevated levels of Mcl-1 which in turn contributes to resistance against the multi-kinase inhibitor regorafenib in colorectal cancer (CRC) patients [63]. Consequently, pharmacological inhibition of Mcl-1 restored the sensitivity of CRC cells to regorafenib in the background of mutant FBW7 [64]. AML patients harboring FMS-like tyrosine kinase-3-internal tandem duplications (FLT3-ITD) exhibit poor outcomes upon treatment with cytarabine- and anthracycline-based induction therapy primarily due to therapy resistance mediated by selective upregulation of Mcl-1. Suppressing Mcl-1 expression restored the sensitivity of AML cells to these targeted therapeutics [65]. The mutant BRAF-driven colon cancer cells depend upon Mcl-1 stabilization to exhibit resistance to the mammalian target of rapamycin (mTOR) inhibitor everolimus [66]. Similarly, BRAF mutation driven melanoma exhibited resistance to BRAF inhibitors vemurafenib or dabrafenib due to overexpression of Mcl-1. Targeted downregulation or pharmacological inhibition of Mcl-1 in combination with the BRAF inhibitors increased the tumor cell killing efficacy [67]. Mcl-1 also contributes to metastasis in breast cancer and resistance to proto-oncogene tyrosine-protein kinase Src (Src) inhibitor dasatinib. Inhibition of Mcl-1 effectively suppressed the metastatic potential of breast cancer cells and increased the overall efficacy of dasatinib [68].

Since interactions between the antiapoptotic and proapoptotic Bcl-2 family proteins are primarily dependent on their BH3 domains, BH3 mimetics were explored as the promising inhibitors of the antiapoptotic Bcl-2 family proteins to trigger apoptosis [69]. One of the earliest BH3 mimetics to be discovered was ABT-737 and its clinical analog ABT-263 (navitoclax) which demonstrated a high affinity towards Bcl-2, Bcl-xl, and Bcl-w [70, 71]. ABT-737 thus exhibited potent single-agent activity against lymphoma, small cell lung cancer, and AML. However, ABT-737 demonstrated little or no activity towards Mcl-1, rendering it refractory. Targeted downregulation of Mcl-1 reinstated the sensitivity of cancer cells towards ABT-737, suggesting that Mcl-1 is a major resistance factor against ABT-737 and its derivatives and that suppressing Mcl-1 expression or its pharmacological inhibition would yield a much better clinical outcome [72–74]. The clinical efficacy of a similar BH3 mimetic that specifically targets Bcl-2, called ABT-199 (venetoclax) was limited due to Mcl-1 overexpression in AML. But combining ABT-199 with an Mcl-1 specific inhibitor S63845 increased the antitumor activity manifold [75]. Further, Mcl-1 inhibition by S63845 demonstrated potent cell kill activity in a wide variety of hematological malignancies and solid tumor cell lines, as a single agent or in combination with other inhibitors [76].

Proteasome inhibitors like bortezomib (velcade) and carfilzomib have been used as an effective therapeutic arsenal in a variety of human cancers [77, 78]. Despite the early success, proteasome inhibitors met with primary resistance in solid tumors and often developed acquired resistance in hematological malignancies, particularly myeloma and mantle cell lymphoma (MCL) after promising initial responses. A variety of adaptive molecular alterations have been cited for the acquired resistance to proteasome inhibitors [79–81]. Since Mcl-1 is a rapid turnover protein with a characteristic short half-life and that it is rapidly degraded by the ubiquitin proteasome system, it is not surprising that proteasome inhibitors would cause stabilization of Mcl-1 protein, thereby contributing to its overall elevated levels and the ensuing therapy resistance. Indeed, Mcl-1 upregulation in response to proteasome inhibitors has been regarded as a major factor to prevent apoptotic cell death in cancer cells, and its targeted downregulation or pharmacological inhibition enhances the cell death induced by proteasome inhibitors [82–84]. Therapy resistance is a distinguishing hallmark of a subset of tumor cells called “Cancer stem cells”. Evidently, the therapy resistance contributed by Mcl-1 has been associated with cancer stem cell-like features in a variety of tumors [29, 32, 85–88]. It is thus evident that Mcl-1 confers tumor cells with resistance against not only conventional therapies but also targeted therapies, contributing to poor clinical outcomes.

### Mcl-1 contributes to therapy resistance by blocking MOMP

**Figure 2.**
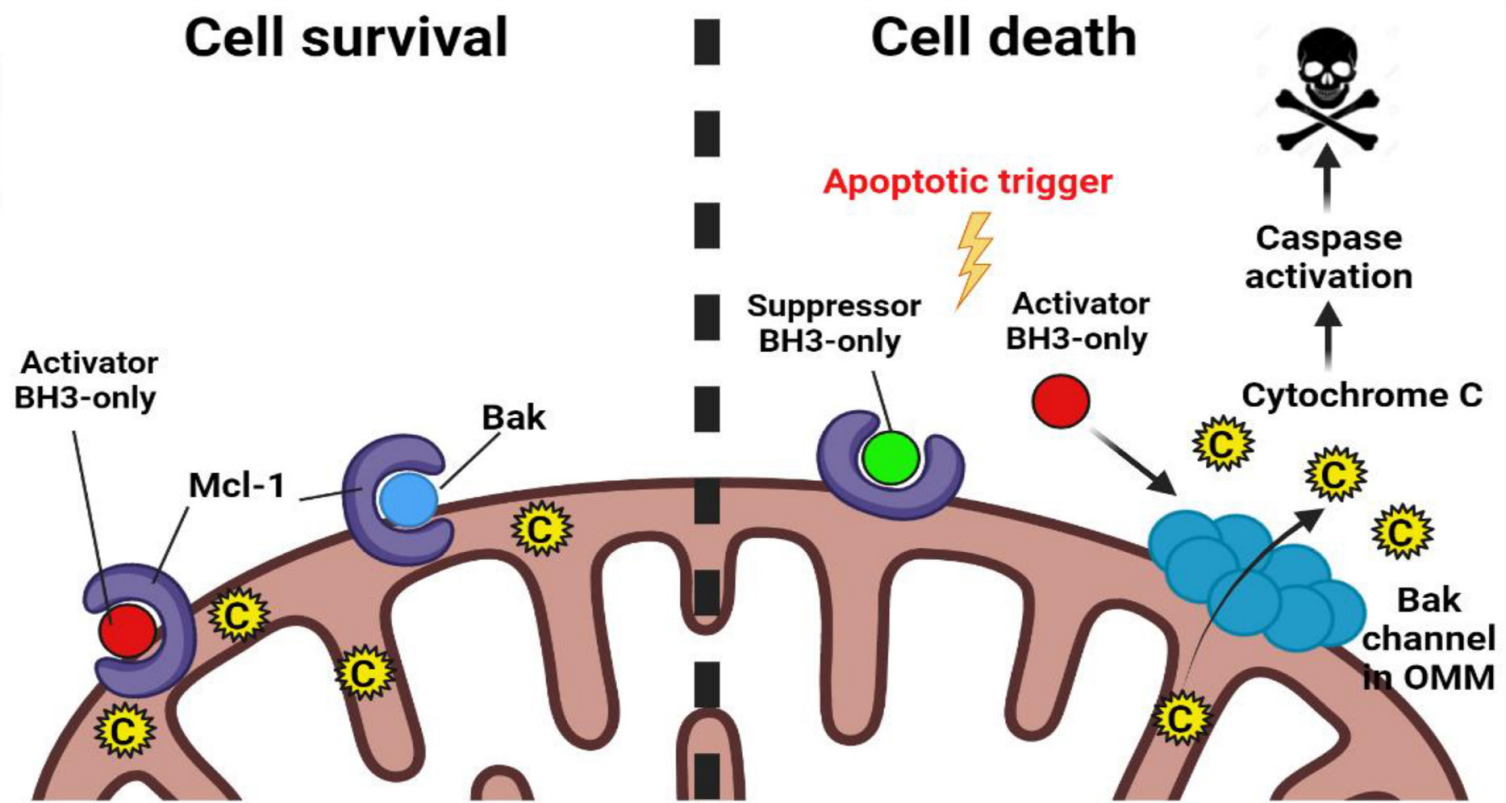
Regulation of apoptosis by Mcl-1. Mcl-1 interacts with “Activator BH3-only” and effector proapoptotic proteins such as Bak in the mitochondrial outer membrane. In response to the apoptotic trigger, Mcl-1 is either rapidly degraded or is antagonized by “Suppressor BH3-only” proapoptotic proteins. This releases Activator BH3-only proteins and Bak from the inhibitory hold of Mcl-1. Activator BH3-only proteins bind to Bak bringing about conformational changes in it and allowing it to oligomerize in the MOM to form hydrophilic channels and release cytochrome C from the mitochondrial intermembrane space out into the cytosol to trigger the apoptotic cascade. C: cytochrome C; OMM: outer mitochondrial membrane. (Figure created with BioRender.com)

The most well-studied mechanism of Mcl-1-mediated therapy resistance in tumors is through the canonical pathway of apoptosis inhibition. On one hand, Mcl-1 interacts with the “Activator BH3-only proteins”—Bid, Bim, Puma thereby disabling their ability to activate the effector proteins of MOMP—Bak, Bax, and Bok. On the other hand, Mcl-1 can directly engage with Bak/Bax, preventing them from oligomerization and blocking MOMP (Figure 2) [8, 12].

**Figure 3.**
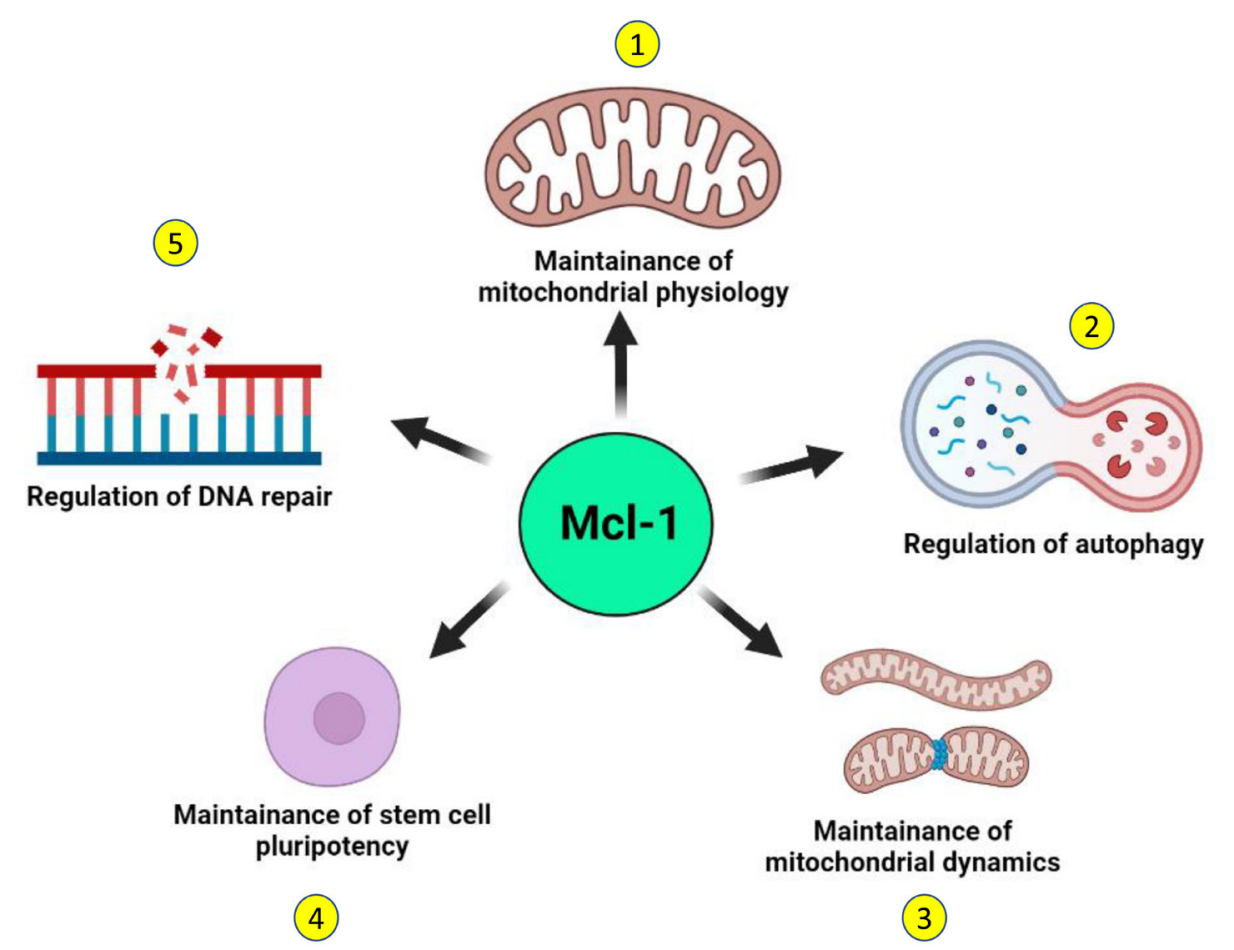
Noncanonical functions of Mcl-1. Independent of its canonical antiapoptotic function, Mcl-1 performs several noncanonical functions in the normal cell that may support the survival of tumor cells. 1. A distinct species of Mcl-1 that localizes to the mitochondrial matrix supports mitochondrial respiration and maintains the organization of the mitochondrial inner membrane. 2. Mcl-1 regulates the induction of autophagy in a developmentally regulated manner and can also induce prosurvival autophagy in response to stress. 3. Independent of its apoptotic functions, Mcl-1 may also regulate the mitochondrial network dynamics through interactions with components of the mitochondrial fusion-fission machinery. 4. Mcl-1 is key not only for the survival of stem cells but also for the maintenance of their stemness. 5. Mcl-1 can actively contribute to DNA repair by interacting with key DNA damage repair proteins and by localizing at the sites of DNA damage. (Figure created with BioRender.com)

Distinct from these canonical functions, recent reports suggest the existence of some noncanonical functions of Mcl-1 that may further contribute to the survival of tumor cells (Figure 3). Mcl-1 has been shown to localize to the sites of DNA damage along with other proteins and mediate DNA repair function [33–35, 89–91]. Apart from the dominant species of Mcl-1 with antiapoptotic function that localizes to the mitochondrial outer membrane, a unique subspecies of Mcl-1 localizes exclusively to the mitochondrial matrix and plays a key role in the mitochondrial respiration [92]. This unique species of Mcl-1 also interacts with the mitochondrial GTPases—dynamin-related protein 1 (DRP1) and optic atrophy 1 (OPA1) and remodels the mitochondrial network. Mcl-1 expression is induced in human pluripotent stem cells in response to reprogramming and its downregulation compromised the remodeling ability of mitochondrial network together with loss of stemness characteristic transcription factors octamer-binding transcription factor 4 (OCT4) and NANOG [87]. Inhibition of Mcl-1 causes mitochondrial fragmentation independent of apoptosis, suggesting that Mcl-1 is important in maintenance of mitochondrial network architecture [88]. Beyond apoptosis, Mcl-1 also regulates autophagy and may promote survival of tumor cells. Mcl-1 also regulates induction of autophagy in response to stress in a developmentally regulated manner [85]. Loss of Mcl-1 was found to be associated with impaired autophagy and it translates to cardiomyopathies [86]. Thus, canonical and noncanonical prosurvival functions of Mcl-1 together support the growth of tumor cells, thereby further contributing to therapy resistance and poor prognosis.

## Therapeutic targeting of Mcl-1

Ever since the prognostic significance of Mcl-1 in tumors was realized, strategies to combat its elevated levels were explored. It mainly involved two approaches: strategies to suppress Mcl-1 expression and direct pharmacological inhibition of Mcl-1 to inhibit its antiapoptotic function.

### Strategies to downregulate Mcl-1 expression

As discussed above, Mcl-1 downregulation often proves to be sufficient to induce apoptosis in a variety of cancer cells. Hence, some of the earliest approaches to target Mcl-1 were aimed at repressing Mcl-1 protein expression.

#### Transcriptional repression of Mcl-1

Although not specifically designed to target Mcl-1, several drugs demonstrated target cell killing activity through Mcl-1 downregulation. Cyclin-dependent kinase (CDK) inhibitors such as flavopiridol is one such class of compounds that has been shown to potently downregulate Mcl-1 expression in B-cell chronic lymphocytic leukemia (CLL) that resulted in patients with high risk and refractory CLL [93, 94]. Similar to flavopiridol, the CDK inhibitor selicliclib potently inhibits RNA polymerase II (RNA Pol II)-dependent transcription leading to a rapid downregulation of Mcl-1 mRNA [95, 96]. The multi-kinase inhibitor sorafenib potently induces apoptosis in cancer cells through a mechanism that involves Mcl-1 downregulation [97, 98]. Signal transducer and activator of transcription proteins 3 (STAT3) and STAT5 bring about transcriptional activation of Mcl-1 [93, 94] and are therefore attractive targets. A variety of natural and synthetic compounds such as resveratrol [98], 2-cyano-3, 12-dioxooleana-1, 9(11)-dien-28-oic acid-methyl ester (CDDO-Me) [97], interferon-α (IFN-α) [99], dasatinib [100], PD180970 [101] and imatinib (STI-571) [102] have shown potent inhibitory effects on STATs, causing potent Mcl-1 downregulation and induction of apoptosis.

#### Destabilizing Mcl-1 mRNA

As opposed to the general inhibitors of the transcription discussed above, antisense oligonucleotides and RNA interference (RNAi) allow specific and targeted downregulation of gene expression by either triggering degradation of the mRNA or blocking the translation. The antitumor potency of Mcl-1 antisense oligonucleotides either alone or in combination with other conventional therapeutics has been demonstrated in a wide variety of human cancers including chronic myeloid leukemia (CML) [94], sarcoma [103], hepatocellular carcinoma [13], gastric cancer [104] and melanoma [105].

#### Modulating the splicing of Mcl-1 mRNA

Alternative splicing of Mcl-1 pre-mRNA is a characteristic feature of Mcl-1 which generates the long isoform (Mcl-1_L_) with antiapoptotic function, a short (Mcl-1_S_) and an extra short isoform (Mcl-1_ES_) both having proapoptotic roles which are predicted to function by interacting with the long isoform and counteracting it [29, 30]. Therapeutic modulation of Mcl-1’s alternative splicing, therefore, offers great promise [106]. Several therapeutic agents are known to shift the balance of Mcl-1 so that there’s a high ratio of Mcl-1_S_/Mcl-1_L_ thereby triggering cell death. Compounds such as epigallocatechin gallate (EGCG) and ibuprofen [13], SDX-101 [105] have been shown to upregulate Mcl-1_S_ with a concomitant decrease in Mcl-1_L_ mRNA levels.

#### Blocking the translation of Mcl-1 mRNA

Mcl-1 mRNA is also characterized by a short half-life similar to Mcl-1 protein which can be used as an advantage to lower Mcl-1 levels in tumors. Several compounds—both natural and synthetic block translation of Mcl-1 mRNA including homoharringtonine [107] and sorafenib [108]. This results in the rapid decline of the Mcl-1 protein levels.

#### Posttranslational targeting of Mcl-1 protein

Since Mcl-1 protein is critical for survival, tumor cells maintain its sustained elevated levels partly through its posttranslational stabilization-deubiquitination. As the deubiquitinase USP9X is known to stabilize Mcl-1 [36, 54], the deubiquitinase inhibitor WP1130 has been shown to induce apoptosis in a manner that involves potent Mcl-1 downregulation [37, 99]. Apart from USP9X, USP13 [38] and USP7 [39] have been implicated in deubiquitination and thereby stabilization of Mcl-1 in various cancer settings. The DNA repair protein Ku70 is also implicated in the stabilization of Mcl-1 and imparting resistance to apoptosis [109]. Inhibiting these Mcl-1 stabilizing mechanisms may enhance the tumor-killing efficacy in Mcl-1-dependent cancer cells.

### Small molecule Mcl-1 inhibitors

#### BH3 mimetics

Since the molecular interactions between the proapoptotic and the antiapoptotic Bcl-2 family proteins, including Mcl-1 are mediated by the BH3 domain of proapoptotic proteins, compounds that mimic the BH3 domain were explored as the first choice for the development of Mcl-1 inhibitors. BH3 mimetic small molecules are predicted to function by displacing the “Activator BH3-only” and multidomain effector proapoptotic proteins—Bak and Bax from the inhibitory hold of antiapoptotic proteins, thereby driving the MOMP. Over the years, a large number of BH3 mimetics with high affinity were developed and evaluated either as pan-Bcl-2 inhibitors or Mcl-1-specific inhibitors against a variety of tumors. These compounds have been outlined in chronological order of their development (Table 2).

**Table 2.** List of select pan-Bcl-2 or Mcl-1 specific inhibitors

**Compound**	**Target proteins**	**References**
**Compounds that down regulate Mcl-1 expression**		
Flavopiridol	CDK	[93, 94]
Seliciclib	CDK	[95, 96]
Sorafenib	Kinases	[108]
Resveratrol	STAT transcription factors	[98]
**BH3 mimetics**		
Pan-Bcl-2 inhibitors		
GX15-070 (obatoclax)	Bcl-2, Mcl-1, Bcl-xl, Bcl-w, Bcl-B	[119–121]
Gossypol (AT-101)	Bcl-2, Mcl-1, Bcl-xl	[122, 123, 140]
BI-97C1 (sabutoclax)	Bcl-2, Mcl-1, Bcl-xl, Bfl-1	[88, 116, 118]
TW-37	Bcl-2, Mcl-1, Bcl-xl	[7, 110, 111]
Mcl-1-specific inhibitors		
MIM1	Mcl-1	[110, 112, 113]
Maritoclax (marinopyrrole A)	Mcl-1	[68, 123, 126]
UMI-77	Mcl-1	[51, 127, 128]
AZD5991	Mcl-1	[17]
S63845	Mcl-1	[76]
AMG-176	Mcl-1	[66, 130, 131]
AMG-397	Mcl-1	[132]

#### BH3 profiling

Due to the complex interaction network of more than 15 Bcl-2 family proteins, it’s challenging to screen and identify novel small molecule BH3 mimetic compounds. A bonafide BH3 mimetic compound must act at the mitochondria by selectively interacting with an antiapoptotic protein with a high affinity and induce MOMP in a Bak/Bax-dependent manner, leading to apoptosis. BH3 profiling enables evaluating the activity of putative BH3 mimetic compounds by analyzing MOMP in terms of cytochrome C release or by the extent of mitochondrial membrane potential (ΔΨ) as a marker for apoptosis [110, 111]. Thus BH3 profiling allows distinguishing between on-target BH3 mimetics from that of the putative mimetics [110]. Moreover, it allows for predicting the effectiveness of therapies in tumors [112, 113]. Thus, BH3 profiling is an invaluable tool in the screening of on-target BH3 mimetics.

#### Pan-Bcl-2 inhibitors

Several pan-Bcl-2 inhibitors have been demonstrated to possess Mcl-1 inhibitory potentials such as AT-101, TW-37, gambogic acid (GA) and sabutoclax (BI-97C1). Our studies with obatoclax (GX15-070), one of the pan-Bcl-2 inhibitors capable of inhibiting Mcl-1, demonstrated a potent single-agent activity against oral cancer cells *in vitro* and *in vivo* by inducing a rather non-canonical form of cell death, namely “necroptosis” [57]. Further, a number of excellent articles have reviewed the recent development and current status in the clinical trials of many of these compounds [104, 108, 109, 114–116].

#### Selective Mcl-1 inhibitors

Although functionally redundant to a certain extent, Mcl-1 is structurally distinct from Bcl-2 or Bcl-xl. The BH3 binding groove of Mcl-1 retains an alpha helix similar to Bcl-2/Bcl-xl but is more electropositive and is flanked by positively charged residues. Additionally, some surface helices of Mcl-1 are positioned differently when compared to Bcl-2/Bcl-xl [117]. These structural differences led to Mcl-1 being refractory to early generation BH3 mimetics such as ABT-737, ABT-263, ABT-199 that efficiently target Bcl-2/Bcl-xl [84]. These structural insights proved to be instrumental in the design of next-generation selective Mcl-1 inhibitors which includes UMI-77; A-1210477; Fesik group (from Vanderbilt University) compound—VU661013; Amgen compounds—AMG176, AMG397; AstraZeneca compound—AZD5991, S64315 and S63845 [118] (Table 3).

**Table 3.** Select clinical trials of Mcl-1-specific inhibitors

**Mcl-1 inhibitor**	**Clinical trial number**	**Phase**	**Intervention**	**Disease**	**Status**
MIK665 (S64315)	NCT02992483	I	MIK665 (S64315)	MM, DLBCL, and lymphoma	Completed
MIK665 (S64315)	NCT04702425	I	MIK665 in combination with Bcl-2 inhibitor VOB560	NHL, AML, and MM	Ongoing
MIK665 (S64315)	NCT04629443	I/II	S64315 plus azacitidine	AML	Ongoing
MIK665 (S64315)	NCT02979366	I	S64315	AML and MDS	Completed
S64315	NCT03672695	I	S64315 plus venetoclax	AML	Ongoing
Gossypol (AT-101)	NCT00390403	I	AT-101 plus temozolomide with or without radiation therapy	Glioblastoma multiforme	Completed
AMG176	NCT02675452	I	AMG176, azacitidine, itraconazole	Relapsed or refractory MM and AML	Ongoing
AMG397	NCT03465540	I	AMG397, azacitidine, dexamethasone	MM, AML, NHL, and MDS	Terminated

DLBCL: diffuse large B-cell lymphoma; MDS: myelodysplastic syndrome. Source: clinicaltrials.gov

#### Allosteric Mcl-1 inhibitors

Beyond the BH3 mimetics, an alternative mechanism for countering Mcl-1 is its allosteric inhibition. Covalent modification of Mcl-1 by a small molecule Mcl-1 allosteric inhibitor molecule 1 (MAIM1) at C286 residue within a noncanonical interaction site that is distant from the BH3 binding groove strongly inhibits the ability of Mcl-1 to bind BH3 domains from the proapoptotic proteins [117, 119].

#### Dual Mcl-1 inhibitors

Due to their partial functional overlap, inhibition of one antiapoptotic Bcl-2 family protein may lead to the overexpression/activation of other members. To overcome the development of potential drug resistance, dual inhibitors are being explored. Based on the structure-guided analyses of the selective Mcl-1 and Bcl-xl inhibitors, a series of hybrid compounds were designed by tethering the Mcl-1 and Bcl-xl inhibitors together. One such optimized compound from the series-compound 11 demonstrated optimal dual inhibitory activity [Mcl-1, half-maximal inhibitory concentration (IC_50_) = 0.088 μmol/L; and Bcl-xl, IC_50_ = 0.0037 μmol/L] [120]. A conserved BimBH3 peptide consisting of 14 residues was modified by inserting two cyclization constraints to generate bicyclic helical peptides, which were potent, cell-permeable, dual inhibitors of Bcl-2A1/Bfl-1 and Mcl-1 with high selectivity over other Bcl-2 proteins [121]. Another study reported the design of dual Mcl-1/Bfl-1 inhibitors based on benzoic acid scaffolds, where a compound 24 binds to both Mcl-1 and Bfl-1 with inhibitor constant Ki of 100 nmol/L and exhibits great selectivity over Bcl-2/Bcl-xl [122]. The natural compound marinopyrrole A has been shown to degrade Mcl-1 protein and thereby induce apoptosis in Mcl-1-dependent cancer cells [123]. The group went ahead and synthesized a series of marinopyrrole A analogs evaluated for their ability to disrupt the interaction between Mcl-1-Bim and Bcl-xl-Bim. Of these, compound 42 was found to potently inhibit the Mcl-1-Bim (IC_50_ = 600 nmol/L) and Bcl-xl-Bim (IC_50_ = 500 nmol/L) interaction, proving it to be a dual inhibitor.

### Targeted degradation of Mcl-1: proteolysis-targeting chimera

Since Mcl-1 is a protein with a short half-life and is stabilized (in addition to being overexpressed) in tumors through a variety of mechanisms, stimulating targeted degradation of Mcl-1 seems a very promising idea. Proteolysis-targeting chimeras (PROTACs) are a novel class of molecules designed to degrade oncogenic proteins where a chemically synthesized small linker serves as a bridge between an E3 ligase and a target protein [124]. Wang et al. [125] reported the development of PROTACs C3 targeting Mcl-1 (DC_50_: 0.7 μmol/L; DC_50_ value represents the concentration where 50% of the target protein-Mcl-1 has been degraded) and C5 targeting Bcl-2 (DC_50_: 3 μmol/L) by conjugating E3 ligase Cereblon (CRBN)-binding ligand pomalidomide with Mcl-1/Bcl-2 dual inhibitors S1-6 and Nap-1. It was demonstrated by an enhanced cell killing efficacy in Mcl-1-dependent H23 cells. PROTAC, therefore, offers a great potential to induce tumor cell death by specifically triggering Mcl-1 degradation and efforts are underway to develop PROTAC probes for Mcl-1 with high sensitivity.

## Conclusions

Owing to the dysregulation of myriad pathways that control Mcl-1 expression, cancer cells of diverse lineages tend to overexpress the antiapoptotic Mcl-1 protein. Elevated Mcl-1 protein levels have been linked with resistance against both conventional as well as targeted therapies in tumors independent of the lineage, contributing to poor clinical outcomes. Although primarily acting through the inhibition of apoptosis, Mcl-1 may also perform its prosurvival functions through mechanisms that are independent of its antiapoptotic role. Although initial efforts to target Mcl-1 in the clinics were curtailed mainly by the toxicities and adverse effects, newer generation Mcl-1 inhibitors are exhibiting better efficacy at the minimal dosage and with a carefully administered dosage regime, will yield better therapeutic outcomes with minimal adverse events.

## Abbreviations

aa: amino acid
AML: acute myeloid leukemia
Bak: B cell lymphoma-2 antagonist killer 1
Bax: B cell lymphoma-2-associated x protein
Bcl-2: B cell lymphoma-2
Bcl-w: B cell lymphoma-2 like 2
Bcl-xl: B cell lymphoma-extra large
Bfl-1: B cell lymphoma-2-related protein A1
BH: B cell lymphoma-2 homology
Bim: B cell lymphoma-2-interacting mediator of cell death
BRAF: B-Raf proto-oncogene
CDK: cyclin-dependent kinase
EGFR: epidermal growth factor receptor
ERK: extracellular signal-regulated kinase
FBW7: F-box and tryptophan-aspartic acid repeat domain-containing protein 7
Mcl-1: myeloid cell leukemia-1
MEK: mitogen-activated extracellular signal-regulated kinase kinase
MM: multiple myeloma
MOM: mitochondrial outer membrane
MOMP: mitochondrial outer membrane permeabilization
mRNA: messenger RNA
NHL: non-Hodgkin lymphoma
PROTACs: proteolysis-targeting chimeras
STAT3: signal transducer and activator of transcription proteins 3
USP9X: ubiquitin specific peptidase 9 X-linked

## Declarations

### Copyright

© The Author(s) 2022.
